# Modularity and Dynamics of Cellular Networks

**DOI:** 10.1371/journal.pcbi.0020174

**Published:** 2006-12-29

**Authors:** Yuan Qi, Hui Ge

**Affiliations:** Whitehead Institute, United States of America

Understanding how the phenotypes and behaviors of cells are controlled is one of the major challenges in biological research. Traditionally, focus has been given to the characterization of individual genes/proteins or individual interactions during cellular events. However, many phenotypes and behaviors cannot be attributed to isolated components. Rather, they arise from characteristics of cellular networks, which represent connections between molecules in cells. We review the recent progress on analyzing the architecture and dynamics of cellular networks. We also summarize how computational modeling yields insight about cell signaling pathways.

The responses of cells to genetic perturbations or environmental cues are controlled by complex networks, including interconnected signaling pathways and cascades of transcriptional programs. The advance of genome technologies has made it possible to analyze cellular events on a global scale. A number of high-throughput techniques, such as DNA microarrays, chromatin immunoprecipitations, and yeast two-hybrid and mass-spectrometry analyses have been applied to cellular systems [1–10]. These experiments have provided first-draft catalogs of essential components, transcriptional regulatory diagrams, and molecular interaction maps for a number of organisms.

In addition to providing a candidate list of biomolecules involved in biological processes, the high-throughput technologies offer unprecedented opportunities to derive underlying principles of how complex cellular networks are built and how network architectures contribute to phenotypes. A series of important questions in this area have been addressed recently (Figure 1). For example, what are the characteristics of cellular network structures that distinguish them from randomly generated networks? Are the network structures relevant for biological functions? If so, are they evolutionarily conserved and how do they evolve? Are some topological patterns preferred at certain times or conditions? These questions are analogous to those asked in the field of genome sequence analysis, such as identifying biologically relevant sequence motifs and domains, investigating the evolutionary conservation between sequences from different species, and understanding temporal or spatial specificities of regulatory sites. In this paper, we survey recent progress on addressing these questions and use mammalian cell signaling as case studies to discuss how computational analyses of networks shed light on specific biological processes.

**Figure 1:**
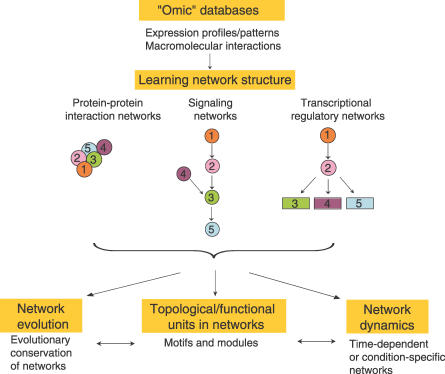
An Overview of Biological Network Analyses Based on “Omic” Data Recent high-throughput technologies have produced massive amounts of gene expression, macromolecular interaction, or other type of “omic” data. Using a computational modeling approach, the architecture of cellular networks can be learned from these “omic” data, and topological or functional units (motifs and modules) can be identified from these networks. Comparisons of cellular networks across different species may reveal how network structures evolve. In particular, the evolutionary conservation of motifs and modules can be an indication of their biological importance. A dynamic view of cellular networks describes active network components and interactions under various conditions and time points. Network motifs and modules can also be time-dependent or condition-specific.

## Modularity of Cellular Networks

**Figure 2:**
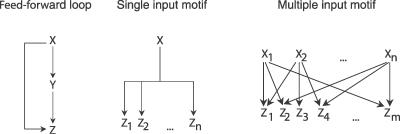
Network Motifs Found in E. coli Transcriptional Regulatory Networks (Left) Feed-forward loop: TF X regulates TF Y, and both X and Y jointly regulate gene Z. (Middle) Single-input motif: TF X regulates genes Z_1_, Z_2_… and Z_n_. (Right) Multi-input motif: a set of TFs X_1_, X_2_… and X_n_ regulate a set of target genes Z_1_, Z_2_… and Z_m_. (Reproduced from [12].)

Unlike random networks, cellular networks contain characteristic topological patterns that enable their functionality. To find the basic building blocks of cellular networks, simple units consisting of a few components were enumerated and some of them were found to be significantly overrepresented [11]. These recurring units were defined as network motifs. For instance, transcriptional network motifs include feed-forward loops, single-input motifs, and multi-input motifs (Figure 2) [3,5,12]. A feed-forward loop describes a situation in which a transcription factor (TF) regulates a second TF, and these two TFs jointly regulate a common target gene. A single-input motif contains one TF which regulates a set of target genes, such as subunits of a protein complex. A multi-input motif consists of multiple TFs that regulate a set of target genes, providing the possibility of combinatorial controls. These motifs are found in multiple organisms such as bacteria, yeast, and human. This structural conservation suggests functional importance of network motifs for transcriptional regulation.

The components of cellular networks, including proteins, DNA, and other molecules, act in concert to carry out biological processes. These functionally related components often interact with one another, forming modules in cellular networks [13]. While motifs represent recurrent topological patterns, modules are bigger building units that exhibit a certain functional autonomy. Modules may contain motifs as their structural components. Modules may maintain certain properties such as robustness to environmental perturbations and evolutionary conservations [13].

**Figure 3:**
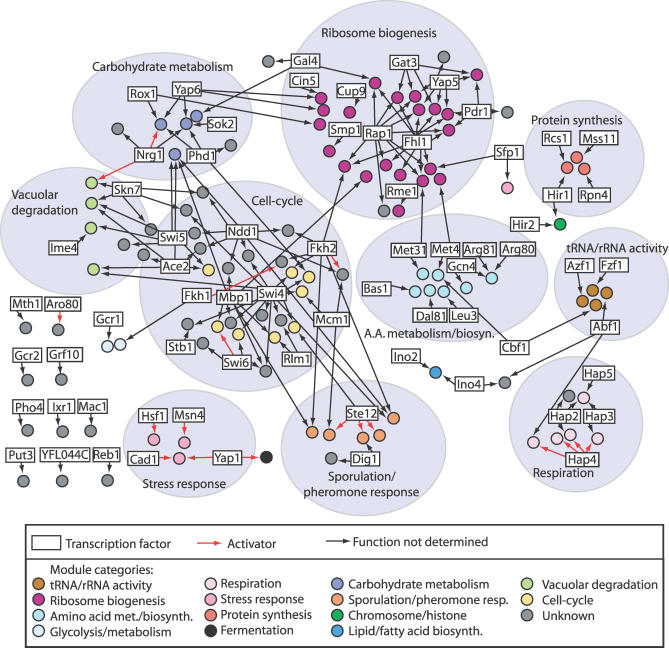
Yeast Transcriptional Regulatory Modules Nodes represent modules, and boxes around the modules represent module groups. Directed edges represent regulatory relationship. The functional categories of the modules are color-coded. (Reproduced from [15].)

Modularity exists in a variety of biological contexts, including protein complexes, metabolic pathways, signaling pathways, and transcriptional programs. For transcriptional programs, modules are defined as sets of genes controlled by the same set of TFs under certain conditions [14]. Gene expression experiments often do not reveal direct regulations. However, if we assume that the expression profiles of regulators provide information about their activities, expression data contains information about regulatory relationships between regulators and their target genes. Bayesian networks, directed probabilistic graphical models (Box 1), were applied to obtain a modular map of Saccharomyces cerevisiae transcriptional regulatory networks based on multiple microarray datasets [14]. Protein–DNA binding data provides direct physical evidence of regulatory interactions. Therefore, combining genome-wide protein–DNA binding data with gene expression data improves the detection of transcriptional modules over using either data source alone (Figure 3) [15]. While each module has a distinct combination of regulators, modules that share regulators can be grouped together [14,15].

Motifs and modules are also found in protein–protein interaction (PPI) networks and metabolic networks [8,9,16–19] (Box 1), which may be indicative of multi-subunit protein complexes or members of metabolic pathways. For these networks, modules can be defined as subnetworks whose components' entities (e.g., proteins or metabolites) are more likely to be connected to each other than to entities outside the subnetworks [19]. For example, recent analyses of affinity purification/mass spectrometry of the yeast proteome identified several hundred novel core complexes and conditional binding modules based on co-occurrence of proteins from multiple purifications [8]. The proteins assigned to the same core complex or binding module tend to share similar temporal expression profiles and subcellular localizations, which supports the functional relevance of modular organization.

The modular organization of cellular networks provides testable hypotheses that lead to biological insights. First, genes in a given module are hypothesized to be functionally coherent. For instance, PPI modules contained proteins involved in common functions such as RNA polyadenylation and chromatin remodeling [17], suggesting strong correspondence between network topology and functionality. Thus, uncharacterized genes or proteins belonging to modules could be functionally annotated accordingly. Second, module structures provide key regulatory information. Using yeast gene expression data, Segal and coworkers [14] inferred regulatory modules that contained regulators and their potential target genes, and predicted conditions under which the regulatory relationships are relevant. The regulatory roles of several previously uncharacterized TFs and signaling molecules were subsequently verified by checking the transcriptional changes of potential target genes upon disruption of regulator functions. For example, Ypl230w, a putative zinc-finger TF, was predicted to play a regulatory role during entry into stationary phase. Ypl230w deletion strain showed no obvious defects under normal conditions. During entry into stationary phase, however, expression levels of predicted Ypl230w target genes changed significantly in the deletion strain compared with normal strains, validating the condition-specific regulatory module. Third, connections between modules highlighted the fact that cellular processes are orchestrated events [14,15,17,20]. For example, connections between glycolysis and lipid metabolism modules revealed their transcriptional coordination [20]. Examination of the target genes in the modules suggested the coupling of glycolysis and phospholipids signaling, which is supported by recent literature.

It should be noted that common assumptions made in the effort to identify modules do not always hold true. In transcriptional module identification, for instance, protein–DNA interactions indicate physical attachment but not necessarily transcriptional activation or repression. Another example is that mRNA expression levels may not effectively reflect TF activities. Systematic profiling of the yeast transcriptome and proteome revealed modest correlation between mRNA expression levels and protein expression levels [21,22]. In addition, post-transcriptional regulation by microRNAs and other noncoding RNAs occurs extensively [23–26], and post-translational modification controls protein activities [10] as well. These effects, once they can be quantitatively determined, should be incorporated into the model.

The error-prone nature and varying scales of high-throughput data increase the difficulty of accurately finding motifs and modules. Current PPI maps may contain a large number of false positives and false negatives. In yeast two-hybrid experiments, for example, proteins are assayed for interactions under nonphysiological conditions. Therefore, the physiological relevance of these interactions is not clear. Recent efforts have categorized or quantified the confidence of two-hybrid interactions [27,28], but the confidence has not yet been used in motif or module finding. Computational approaches that employ probabilistic structure priors of degree distributions [29] or integrate additional types of “omic” data [30] have also been applied to de-noise PPI maps.

## Modules in Evolution

The organization of cellular networks can be examined from an evolutionary perspective. Investigations of PPI networks revealed that proteins belonging to fully connected subgraphs are more likely to be evolutionarily conserved than randomly selected proteins [18]. In return, evolutionary conservation can help to identify modular structures and reveal undescribed functionality and interactions. Sharan and coworkers [31] integrated PPI networks with sequence data to find network regions that were conserved across multiple species. In these conserved regions, novel PPIs were predicted for yeast, and a significant proportion were experimentally verified. These PPIs would not have been found by investigating networks in a single species alone.

Module evolution of transcriptional regulatory programs has also been probed. In an analysis of expression profile compendia, Stuart and coworkers [32] defined metagenes as sets of orthologs in multiple species. Metagenes coexpressed across species were more likely to be functionally related than those coexpressed in any single species. Based on this notion, functional modules were constructed by clustering coexpressed metagenes [32] (Box 1). The cell proliferation module, for example, contained genes that were not previously known to be involved in this process. Five of them were subjected to experimental tests, and the results provided supportive evidence for their roles in cell proliferation. Though transcriptional modules are conserved across species, Tanay and coworkers [33] showed that cis-regulatory elements controlling gene expression of some conserved modules might have diverged during evolution. By comparative genomics analysis, they suggested an intermediate redundant regulatory program, which enabled a gradual switch from one regulatory program to another while maintaining functionality. Such hypotheses are still to be verified by additional experimental data. Protein–DNA binding data for TFs across different species will provide evidence on the extent to which the regulatory programs are conserved, and whether intermediate programs exist during the evolution of transcriptional regulation. The study of conserved modules from multiple species can potentially elucidate how relevant biological functions are kept in modules while individual genes may have acquired new properties during evolution.

## Cellular Networks as a Dynamic System

A living cell is a dynamic system, where gene activities and interactions exhibit temporal profiles and spatial compartmentalizations. Interactions presented in a static network may not necessarily occur simultaneously. A typical example is Cdc28p, a cyclin-dependent kinase with a constant expression profile, which interacts with a variety of cyclins at different phases of the cell cycle [34]. Dynamic descriptions of networks are necessary for an accurate understanding of cellular events. By integrating yeast PPI networks with gene expression data, Han and coworkers [35] suggested that some modules are active at specific times and locations. In a study that described dynamic protein complex formation during cell cycles [34], it was found that constitutively expressed and cell cycle–regulated proteins together form protein complexes at particular time points during the cell cycle. This suggested a general mechanism of “just-in-time-assembly,” where only some subunits of protein complexes are regulated during cell cycle progression and the synthesis of these subunits control the timing of complex assembly. “Just-in-time-assembly” may be a more efficient way of regulation compared with “just-in-time-synthesis,” in which case all subunits of protein complexes are regulated and synthesized at the same time during the cell cycle.

**Figure 4:**
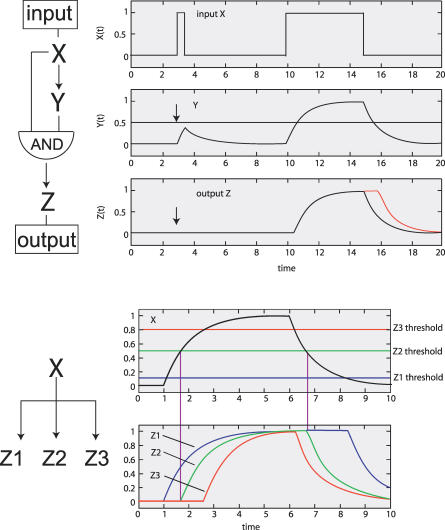
Dynamic Properties of Network Motifs (Upper panels) Shows a feed-forward loop, where Y is an accumulation of X over time, and the product of X and Y passes a threshold (thin horizontal line) to activate Z. This loop rejects impulsive perturbations in X, and responds only to persistent activation. This is because Y increases gradually to pass the threshold. A similar rejection of impulsive fluctuations can be achieved by a feed-forward chain, where X activates Y and Y activates Z. However, a feed-forward chain responds slower (thin red curve) to the off signal than to the loop. (Lower panels) Shows a single-input motif, where X regulates Z1, Z2, and Z3 (*n* = 3). When X changes over time, Z1, Z2, and Z3 are activated and deactivated in order, based on their thresholds. In particular, Z1, which has the lowest threshold, is activated first and deactivated last. (Reproduced from [12].)

Network topologies reveal dynamic properties that contribute to cellular functions. Though network motifs are generally overrepresented in static transcriptional networks, the frequency of presence for each motif type varies under different conditions. By integrating TF binding data with gene expression data, Luscombe and coworkers [36] constructed condition-specific transcriptional subnetworks for yeast, and these subnetworks each showed preference for certain types of network motifs, highlighting the different dynamic properties required for each condition. Specifically, “endogenous” subnetworks favored feed-forward loops which are suitable for keeping long-lasting signals to drive multi-staged, endogenous processes, such as the cell cycle, while removing sporadic noise. “Exogenous” subnetworks favored single-input motifs which are suitable for initiating a quick and coordinated response to external stimuli (Figure 4). The condition-specific preference of network motifs also suggests that even though motifs may be used as building blocks to reconstruct regulatory networks, caution should be taken in bottom-up reconstruction efforts, since the building blocks may vary according to the biological functions.

**Figure 5:**
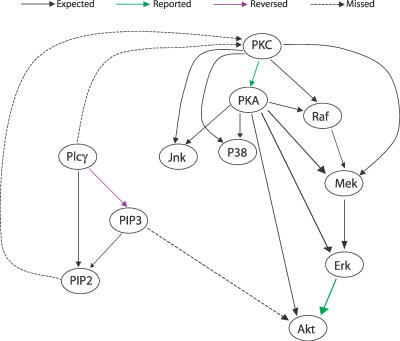
Bayesian Network Modeling of Molecular Interactions in Cell Signaling Nodes in the network represent key signaling molecules. Directed edges represent predicted causal relationships between signaling molecules. Edges are categorized into different classes: (i) well-established interactions in the literature (“expected”); (ii) interactions that have been reported but weakly supported (“reported”); (iii) well-established interactions that Bayesian networks failed to predict (“missing”); (iv) predicted causal relationship in a direction opposite to the literature (“reversed”). (Reproduced from [39].)

Time-series or condition-specific data are required for further in-depth understanding of cellular dynamics. Currently most of these data come from mRNA expression, which is not fully correlated with protein activities [21,22]. Also, these data often reflect composition of cell populations that may not be well-synchronized. More advanced technologies for single cells could significantly propel research in this area [37]. Computationally, general graphical models such as dynamic Bayesian networks may be applied to analyze the dynamics of cellular network structures.

**Box 1. Summary of Computational Methods in Network Modeling Using “Omic” Data**

(a) Clustering

**Figure 6:**
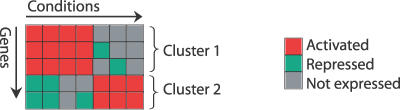
Clustering Methods Genes that share similar expression profiles across conditions are grouped together by clustering.

Clustering methods are widely used to find modules in transcriptional regulation. An expression profile dataset can be represented as a two-dimensional matrix where rows index genes and columns index experimental conditions. Clustering methods partition genes into groups such that genes in each group show similar expression across conditions or through a time series [47] (Figure 6). Since regulation by common TFs may only occur under certain conditions, bi-clustering methods [48] have been developed to identify genes that express similarly under a subset of conditions. It should be noted that genes with similar expression may not all be co-regulated, and that clustering does not necessarily identify the corresponding regulators. Therefore, genes clustered together may not fully represent modules in transcriptional regulatory networks.

Traditional clustering methods, such as K-means, require a predefined and fixed number of gene clusters, which may be hard to assign in practice and greatly influence the results. They also do not model temporal dependence between expression profiles. To address these issues, Schliep and coworkers [49] and Beal and Krishnamurthy [50] applied Hidden Markov Models to cluster gene expression time course data. Specifically, both of them used Hidden Markov Models to model temporal dependence of gene expression, instead of treating different time points independently. While Schliep and coworkers proposed a heuristic approach to determine the number of clusters, Beal and Krishnamurthy used a nonparametric prior distribution on mixture weights, such that the genes can be clustered without a predefined number of clusters.

(b) Topology-based analysis

**Figure 7:**
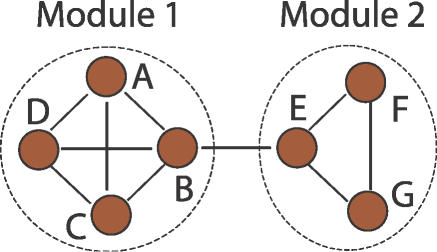
Topology-Based Network Analysis Densely connected subgraphs can be identified from interaction networks, suggesting the existence of multi-component complexes.

Interaction networks are often visualized as graphs where nodes represent genes/proteins and edges represent interactions. Modular structures can be inferred based on topological features of the networks. For example, densely connected subgraphs can be exhaustively identified in PPI networks (Figure 7). These suggest the existence of multi-protein complexes [16]. Also, modules can be identified using topological distances in the networks. More specifically, the distance between two nodes is defined as the length of the shortest path(s) between them. A matrix of distances between all pair-wise combinations of nodes can be used for clustering [17]. The underlying assumption is that proteins in a module have similar distances to proteins outside of the given module.

(c) Probabilistic graphical models

**Figure 8:**
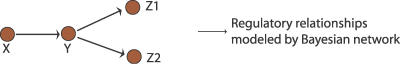
Probabilistic Graphical Models Directed acyclic graphical models are called Bayesian networks. In the shown Bayesian network, values of variable *Y* depend directly on values of *X,* and values of variable *Z1* and *Z2* depend directly on values of *Y*.

Nodes of probabilistic graphical models represent variables, and edges represent independency relations among the variables (Figure 8). According to the directionality of edges, graphical models can be classified into two major categories: Bayesian networks and Markov random fields. A Bayesian network is a directed acyclic graphical model: if there is an edge from node *X* pointing to another node *Y,* then values of variable *Y* depend directly on values of *X* and *X* is called a *parent* of *Y*. Coupled with intervention data, Bayesian networks can be used to learn causal relationships, and are thus suitable to model transcriptional regulatory networks [14,51,52] or signaling pathways [39]. In contrast, the edges in Markov random fields are undirected, which makes them suitable to model PPI networks or other networks of symmetric interactions [53].

To use graphical models, we need to systematically learn the structures of networks based on biological data and to estimate the parameters of these networks [54]. The learned graphical models reveal how proteins and genes interact, which can be applied to answer different biological queries as an inference problem. For example, when the activities of a protein are suppressed, cells may respond by changing the expression levels of other genes. Such responses can be predicted based on a learned regulatory network.

While the task of learning Bayesian networks has been well-addressed [51,55], learning Markov random fields is still in its early stage [56,57]. If we use graphical models to model large-scale biological networks containing structural loops such as PPI networks, the inference problem is not trivial. Monte Carlo methods or approximate inference methods can be used to solve such problems [55,58–62].

(d) Integration of various data sources

**Figure 9:**
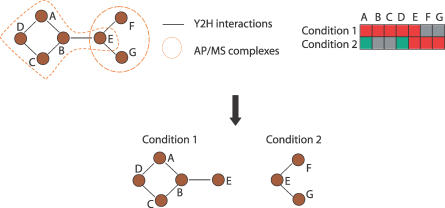
Integration of Multiple Datasets The integration of a variety of datasets, including binary interactions, protein complexes, and expression profiles enables the identification of subnetworks that are active under certain conditions.

Individual high-throughput biological datasets are usually both incomprehensive and error-prone. Therefore, data integration becomes indispensable in order to model cellular networks accurately and to make functional inferences [45]. For example, both yeast two-hybrid [63,64] and affinity-purification/mass-spectrometry experiments [8,9] have been applied to the mapping of PPI networks. Overlapping the two data sources enables the identification of high-confidence interactions [65]. In addition, yeast two-hybrid detects binary relationships while affinity-purification/mass-spectrometry detects proteins as members of a complex. Integrating these two types of data helps to model the actual topology of protein complexes [66]. Furthermore, if temporal, spatial, or conditional expression data are available, it may be possible to provide a dynamic view of protein complexes under physiological conditions (Figure 9).

## Understanding Cell Signaling from a Network Perspective

Having reviewed recent progress in learning the global architecture of cellular networks, we proceed to discuss mammalian cell signaling as a case study where computational models provided specific biological insights. Signaling pathways can be viewed as a module where multiple inputs take their effects through intertwined networks to produce multiple outcomes. Motifs such as feed-forward loops and feedback loops are also enriched in signaling networks, and these motifs affect information propagation of the specific biological process [38]. In a system that is not fully characterized, connections between cellular components can be derived as a first step to understanding how the signaling pathways are wired. To this end, Sachs and coworkers [39] measured phosphorylation states of key signaling molecules in single cells under a variety of conditions. A Bayesian network was constructed to elucidate the causal relationships between these key molecules (Figure 5). The predicted relationships recaptured most of the well-established interactions and contained several causal relationships that were only weakly supported previously. These causal relationships were subsequently confirmed by experiments.

Based on experimental data about signaling pathways, is it possible to predict the responses and behaviors of cells? Janes and coworkers [40] explored this by modeling signal transduction leading to the apoptosis/survival decision switch. Data inputs included the kinase activities and phosphorylation states of signaling proteins over a time course; outputs consisted of a variety of indications for apoptosis. A computational method, partial least squares regression, which models the relationship between inputs and phenotypic outputs, accurately predicted the apoptotic outcomes under previously untested conditions. The pro-apoptotic and anti-apoptotic roles of signaling molecules were correctly inferred from the model. Some signaling molecules may play seemingly self-contradictory roles in apoptosis. By taking dynamic data as inputs, the model accounted for such differential effects of MAPK-activated protein kinase 2 at different time points.

These model-driven approaches should complement hypothesis-driven approaches in making novel discoveries about signaling pathways. Despite exciting progress, much remains to be improved in modeling cell signaling. One general concern is that conclusions drawn from these analyses are highly dependent on the modeling assumptions. For example, the apoptosis prediction model assumed a linear relationship between cytokine inputs and phenotypic outputs, while biological systems are often nonlinear [40]. On the experimental side, traditional approaches to identify protein post-translational modification can be time-consuming and thus limit the rate and scale of data generation. Recent advances in proteomic technology allow the identification of phosphorylation states in a high-throughput manner [41–44]. This may enable the model-driven approaches to be applied to many more modules.

## Conclusion

Modularity and dynamics both underlie the functionality of cellular networks, ranging from transcriptional regulation to cell signaling. Technological innovations in both data generation and computational methods may advance our understanding significantly. Furthermore, integrating currently available data from various sources helps us to gain a more accurate and comprehensive understanding of cellular processes [45,46] (Box 1). Currently, the data quality and coverage of high-throughput datasets impose limitations on inferring accurate networks. Many computational methods used for analyzing biological systems do not make full use of available data and/or make strong assumptions that might not be realistic. With progress toward solving these problems, the phenotypes and behaviors of cells could potentially be predicted with higher confidence, and we might realize the promise to re-engineer cellular networks to produce desired properties. 

## Abbreviations

PPI: protein–protein interaction
TF: transcription factor
